# Histopathological evaluation in post-mortem renal biopsies of patients with COVID-19 and comorbidities: a case-control study

**DOI:** 10.1590/1516-3180.2024.0376.R1.13102025

**Published:** 2026-01-19

**Authors:** Samya Hamad Mehanna, Mayara Pezzini Arantes, Henrique Machado de Souza Proença, Rafael Weissheimer, Sérgio Ossamu Ioshii, Micheli Ito Gimenes Pires, Julia Wolf Barretto, Eduardo Morais de Castro, Thyago Proença de Moraes, Lucia de Noronha

**Affiliations:** I PhD student, Escola de Medicina, Programa de Pós-Graduação em Ciências da Saúde, Pontifícia Universidade Católica do Paraná (PUC-PR), Curitiba (PR), Brazil.; II Escola de Medicina, Programa de Pós-Graduação em Ciências da Saúde, Pontifícia Universidade Católica do Paraná (PUC-PR), Curitiba (PR), Brazil.; III Departamento de Patologia, Hospital do Rim, Universidade Federal de São Paulo (Unifesp), São Paulo (SP), Brazil.; IV Ciências da Saúde, Pontifícia Universidade Católica do Paraná (PUC-PR), Curitiba (PR), Brazil.; V Escola Politécnica, Programa de Pós-Graduação em Tecnologia da Saúde, Pontifícia Universidade Católica do Paraná (PUC-PR), Curitiba (PR), Brazil.; VI Hospital Universitário Cajuru, Pontifícia Universidade do Paraná (PUC-PR), Curitiba (PR), Brazil.; VII Escola de Medicina, Faculdades Pequeno Príncipe, Curitiba (PR), Brazil.; VIII Instituto de Pesquisa Pelé Pequeno Princípe; Escola de Medicina, Faculdades Pequeno Princípe, Curitiba (PR), Brazil; IX Pontifícia Universidade do Paraná (PUC-PR), Curitiba (PR), Brazil.; X Pontifícia Universidade do Paraná (PUC-PR), Curitiba (PR), Brazil.

**Keywords:** Biopsy, COVID-19, Diabetes mellitus, Essential hypertension, Acute kidney injury, SARS-CoV-2, Acute tubular necrosis, Diabetic nephropathy, Arterial hypertension, Histopathology, Acute renal failure

## Abstract

**BACKGROUND:**

Acute kidney injury is one of the main systemic complications of severe coronavirus disease 2019 (COVID-19).

**OBJECTIVES:**

To examine histopathological changes in post-mortem kidney biopsies of patients who died as a result of the disease caused by SARS-CoV-2 (Severe Acute Respiratory Syndrome Coronavirus 2).

**DESIGN AND SETTING:**

A case-control study was conducted at a tertiary hospital located in Curitiba, Paraná, Brazil.

**METHODS:**

The study group, called "COVID," consisted of kidney biopsy samples obtained from deceased patients with COVID-19, with a "Control" group included for comparison. Samples were selected based on sex, age, and comorbidities, with an emphasis on diabetes mellitus and systemic arterial hypertension (SAH). Morphological evaluation was performed by pathologists using preestablished criteria with glomerular, tubular, and vascular characteristics among the parameters.

**RESULTS:**

Tubular atrophy and interstitial fibrosis, markers of chronic kidney injury, were observed with equal frequency in both groups, probably because of the initial pairing of the samples. These findings are in line with what would be expected from chronic exposure to proteinuria. In relation to SAH, the main identification was interstitial vascular damage, particularly arteriolosclerosis/arteriosclerosis. Acute tubular injury was the most frequently observed feature in patients in the COVID group, which was probably related to ischemic damage.

**CONCLUSION:**

This study demonstrated that the main change identified in the renal parenchyma of patients with COVID-19 was acute tubular injury, which was expected considering the context of severe systemic ischemia to which these patients are subjected, with the other findings being the consequences of chronic damage.

## INTRODUCTION

 The Severe Acute Respiratory Syndrome Coronavirus 2 (SARS-CoV-2) virus emerged in the last quarter of 2019, rapidly spreading worldwide.^1^ Infection has been documented in various clinical presentations, ranging from asymptomatic infections to fatal cases requiring prolonged treatment in intensive care units.^2,3^ Among the main systemic complications of severe cases, studies highlight interstitial lung disease, thromboembolic events, encephalopathy, and cardiopathies.^4-7^

 Besides the respiratory and cardiovascular systems, patients with coronavirus disease 2019 (COVID-19) may also experience impairment of renal function, with an incidence ranging from 0.5% to 28%.^8^ There is a correlation between the onset of acute kidney injury (AKI) and the need for mechanical ventilation support, with some patients requiring renal replacement therapy.^9^ Furthermore, laboratory tests showed abnormalities such as albuminuria, hematuria, elevated urea, and high levels of creatinine.^10^

 The histological findings of renal biopsies in patients with COVID-19 show that acute tubular injury is the most prevalent outcome, likely related to ischemic damage to the renal parenchyma.^11,12^ However, the presence of the virus directly affecting kidney tissue has not yet been proven,^9^ and further studies are needed to elucidate the worse prognosis of patients with renal injury associated with SARS-CoV-2 infection and to better understand the pathophysiology of the disease. 

 This study aimed to describe glomerular, tubular, and vascular histopathological alterations in post-mortem renal biopsies of patients who died because of COVID-19. Additionally, these findings were compared with those of renal tissues from a properly matched control group. 

## METHODOLOGY

 This case-control study comprises the study group, termed the "COVID group" (n = 17), with renal biopsy specimens preserved in paraffin blocks. Samples were obtained from patients who died owing to complications of different natures caused by COVID19; however, they did not require dialysis when monitoring AKI. Minimally invasive *post-mortem* renal biopsy was performed through percutaneous puncture of the flanks, following appropriate authorization from family members who signed informed consent forms. This study was approved by the Ethics Committee of the Pontifícia Universidade Católica do Paraná (PUC-PR), CAAE: 30188020.7.1001.0020, opinion number 3.944.734. 

 The following criteria were defined for the inclusion of biopsies in the study: patients admitted to the intensive care unit (ICU) of a tertiary hospital located in Curitiba, Paraná, aged ≥ 18 years, with immunological and molecular tests confirming COVID-19 infection, and who died because of the disease. Patients who tested negative (PCR and serologies) for COVID-19 were excluded from the study despite meeting the criteria. 

 The "Control group" (n = 16) consisted of samples from patients matched in sex and comorbidities to the "COVID group," in a ratio of 1:1 to 2:1, obtained from a biological sample archive maintained by a reference hospital, located in São Paulo, collected from surgical specimens of patients undergoing kidney transplantation ("zero time" biopsy). These samples date back to procedures performed before the onset of the pandemic. 

**Table 1** summarizes the data regarding sex, age, and comorbidities of the biopsied patients comprising the final sample, comparing both groups. 

**Table 1:** Comparative table of sex, age, and comorbidities between the Control and COVID groups

**Data**	**Variable**	**Group control**	**Group COVID**
Sex	Male	50% (n = 8)	53% (n = 9)
	Female	50% (n = 8)	47% (n = 8)
Age (years) mean/median (min–max)		51,4/56,5	74,5/78
		(30–72)	(59–86)
Comorbidities	Diabetes mellitus	50% (n = 08)	29,4% (n = 5)
	Systemic arterial hypertension	50% (n = 08)	70,6% (n = 12)

 All kidney samples were fixed in formalin and embedded in paraffin (FFPE), subjected to routine processing, inclusion, cutting at 4 μm, and staining with hematoxylin and eosin, and then taken for microscopic analysis. For a more detailed assessment, the slides were stained with periodic acid-Schiff (PAS) with diastase, Masson’s trichrome, and methenamine silver (PAMS). 

 Morphological evaluation of the biopsies was conducted by three independent pathologists based on pre-established criteria to ensure objectivity. All samples were reviewed by the same team, which helped minimize intra-observer variability and ensure consistency in assessment. The parameters utilized were selected based on the Banff Classification Reference Guide for the evaluation of kidney graft biopsies.^12^ All the criteria stipulated for the evaluation are described in **Table 2**. The Banff Classification was designed for the evaluation of renal graft biopsies to diagnose and grade rejections. We chose to use this classification, even in the context of native samples (not transplants), because the classification parameters evaluated all renal compartments (glomeruli, tubules, interstitium, and vessels). The evaluators were not exposed to clinical data or sample identification before or during the assessment. 

**Table 2:** List of parameters and criteria used in the morphological evaluation of renal biopsies

**Morphological Parameter**	**Evaluation criterion**
Presence of microthrombi	1 = present or 0 = absent
Duplication of the glomerular basement membrane	1 = present or 0 = absent
Glomerulitis	1 = present or 0 = absent
Acute tubular injury	0 = no changes; 1 = mild changes; 2 = moderate changes; and 3 = severe changes
Tubulitis	0 = no foci; 1 = 1 to 4 foci; 2 = 5 to 10 foci; and 3 ≥ 10 foci or focus of tubular basement membrane destruction
Tubular atrophy	0 = No foci; 1 ≤ 25%; 2 = 26 to 50%; 3 > 50%
Tubulointerstitial inflammation in non-atrophic region	0 ≤ 10%; 1 = 10 to 25%; 2 = 26 to 50%; and 3 > 50%
Interstitial fibrosis	0 ≤ 5%; 1 = 6 to 25%; 2 = 26 to 50%; and 3 > 50%
Vasculitis	1 = present or 0 = absent
Arteriosclerosis in cross-section, using the most affected vessel	0 = no foci; 1 ≤ 25% of vessel thickness; 2 = 26 to 50% of vessel thickness; and 3 > 50% of vessel thickness
Arteriolosclerosis in sample	0 = no foci; 1 = mild to moderate in at least one arteriole; 2 = moderate to severe in one arteriole; and 3 = Moderate to marked in several arterioles

 The categorized variables were presented as frequency and simple percentages of the obtained data. The "Control" and "COVID19" groups were compared using Fisher’s exact test. Values of P ≤ 0.05 indicated statistical significance. The data were analyzed using the IBM SPSS Statistics v.28.0 software (Armonk, New York). 

## RESULTS

 The results of the morphological parameter evaluation found in the samples of the "Control" and "COVID" groups are presented in **Table 3**. Fisher’s exact test revealed no statistically significant differences between the two groups, with all P values exceeding 0.05 and 95% confidence interval (95% CI). 

**Table 3:** Morphological variables evaluated comparing the "Control" and "COVID" groups

**Variable**	**Classification**	**Groups**				P^*^
		**Control**		**COVID**		
		**n**	**%**	**n**	**%**	
Presence of microthrombi in glomerulus	Absent	16	100%	17	100%	1
Duplication of glomerular basement membrane	Absent	16	100%	17	100%	1
Expansion of mesangial matrix	No foci	16	100%	17	100%	1
Mesangial cellularity	Up to 4 cells	16	100%	17	100%	1
Glomerulitis	Absent	7	43,8%	6	35,3%	0,73
	Present	9	56,3%	11	64,7%	
Signs of acute tubular injury	Mild to moderate alteration	13	81,3%	17	100%	0,1
	Severe alteration	3	18,8%	0	0%	
Tubulitis	No foci	15	93,8%	16	94,1%	1
	1–10	1	6,3%	1	5,9%	
Tubular atrophy	No foci	11	68,8%	9	52,9%	0,48
	≤ 50%	5	31,2%	8	47,1%	
Tubulointerstitial inflammation in non-atrophic region	< 10%	15	93,8%	14	82,4%	0,6
	≥ 10%	1	6,3%	3	17,6%	
Interstitial fibrosis	≤ 5%	12	75%	7	41,2%	
	6%–50%	4	25%	10	58,8%	0,08
Arteriosclerosis in most affected interstitial vessel	No foci	3	18,8%	6	35,3%	0,44
	0%–50%	13	81,2%	11	64,7%	
Arteriolosclerosis in sample	No foci	12	75%	13	76,5%	1
	Some foci	4	25%	4	23,5%	
Vasculitis	Absent	16	100%	16	94,1%	1
	Present	0	0%	1	5,9%	

* Fisher’s exact test, with significance set at P < 0.05.

 For the variables applied to the morphological evaluation of glomeruli, such as microthrombi, duplication of the glomerular basement membrane, expansion of the matrix, and increased mesangial cellularity, neither the control nor the experimental groups showed alterations. Glomerulitis was identified in 64.7% and 56.3% of patients in the study and control groups, respectively, with no statistically significant differences. It was also observed that there was a prevalence of findings related to chronic organ damage in both the COVID group and the control group (without statistically significant differences), indicating a similar epidemiological profile between the groups, likely due to matching by comorbidities and age, causing an overlap of findings such as tubular atrophy, interstitial fibrosis, and arteriolosclerosis. 

**Figure 1:**
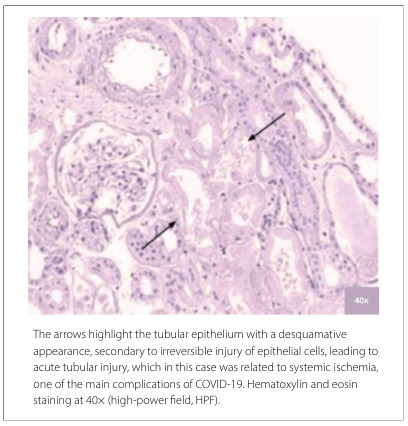
Tubular morphological assessment of renal biopsies from the COVID group.

**Figure 2:**
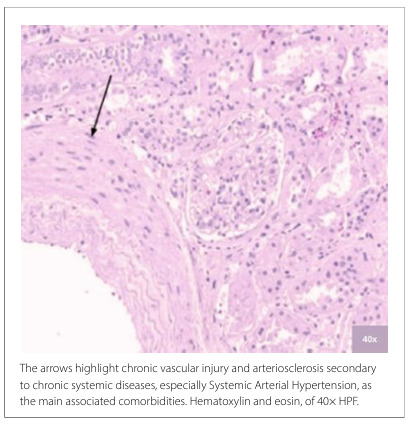
Vascular morphological assessment of renal biopsies from the COVID group.

 Notably, in both groups, all cases presented with acute tubular injury to some degree, albeit not commonly associated with tubulitis, which was observed in only two cases across both groups. Regarding the evaluation of blood vessels in the interstitium, only one case in the COVID group showed changes consistent with vasculitis. However, when considering other vascular alterations, arteriolosclerosis was the most frequently identified, occurring in 81.2% of the cases in the control group and 64.7% of the cases in the COVID group. **Figures 1** and **2** illustrate these morphological findings. 

## DISCUSSION

 COVID-19 disproportionately affects patients with chronic comorbidities, given the accumulated tissue damage in target organs.^2,3^ Consequently, the impact of chronic diseases on the renal parenchyma is expected, with these being the main morphological findings found in the research. The evaluated parameters that indicated the chronicity of the injury were tubular atrophy, interstitial fibrosis, and arteriosclerosis.^13^

 Furthermore, the results corroborate those described in the literature when acute tubular injury secondary to hypoxia was identified in all biopsies of the "COVID" group.^14,15^ Notably, acute tubular injury is not specifically attributed to SARS-CoV-2, but rather to pathological conditions that include tissue oxygen deprivation among their complications. Tubular injury is the most common cause of AKI and is secondary to events that lead to irreversible damage to renal tubular cells and injury to these structures. Among the causes of acute tubular injury, ischemic events are the main triggering factors; however, other causes, including drug-induced nephrotoxicity, may also be associated. The "COVID" group probably presented injuries secondary to hypoxia caused by the infection, since no medications with a high probability of nephrotoxicity were identified in the medical records. Furthermore, signs of acute tubular injury were present in both samples without statistically significant differences, which may be justified based on acute situations involving renal ischemia in the control group (undergoing transplantation), which occurs more easily in patients with the comorbidities.^9,16^

 Regarding the findings related to chronic diseases present in both matched groups, arteriosclerosis stands out as an expected event for patients with comorbidities such as systemic arterial hypertension (SAH) and diabetes mellitus (DM), as it is commonly associated with systemic vascular damage, compounded by the average age of the patients in the sample, most of whom were elderly.^17,18^

 Regarding DM, the literature highlights that the primary initial damage to the renal parenchyma is the thickening of the basal membranes lining the glomerular capillaries and tubules, secondary to the increase in the concentration of proteins in the glomerular ultrafiltrate.^19,20^ Conversely, tubular atrophy and interstitial fibrosis are markers of chronic and prolonged exposure to proteinuria, characteristically observed in patients with diabetes with chronic and advanced renal injury.^21^ These latter two morphological aspects were found with equal frequency in both groups of our study as a result of the initial matching of samples. 

 In the context of SAH, the main histopathological finding is interstitial vascular damage, secondary to increased intravascular pressure and hyperstimulation of the Renin-Angiotensin Aldosterone System, often observed in these patients. Thus, chronic injury to the structural layers of the vessel walls, arteriolosclerosis/arteriosclerosis, is the main histopathological alteration in these renal samples.^22^ Therefore, it was a frequent finding in the research and in both groups, without statistical differences, also resulting from the matching of the sample by comorbidities.^17^

## CONCLUSION

 The results demonstrate that the primary damage to the renal parenchyma identified in patients with severe COVID-19 is acute tubular injury, which is an expected outcome considering the context of severe systemic ischemia to which these patients are subjected. Other findings include the consequences of chronic damage caused by preexisting comorbidities. 

## Data Availability

No new data were generated or analyzed in this study
